# EEG study of the cortical representation and classification of the emotional connotations in words

**DOI:** 10.1186/1471-2202-15-S1-P81

**Published:** 2014-07-21

**Authors:** Yuqiao Gu, Massimo Poesio, Brian Murphy

**Affiliations:** 1 CLIC, CIMeC - Center for Mind/Brain Sciences, Università degli Studi di Trento, Rovereto (TN), I – 38068, Italy; 2 School of Computer Science and Electronic Engineering, University of Essex, Colchester, CO7 9QZ, UK; 3 Knowledge & Data Engineering (EEECS) Queen's University Belfast, UK

## 

**Figure 1:**
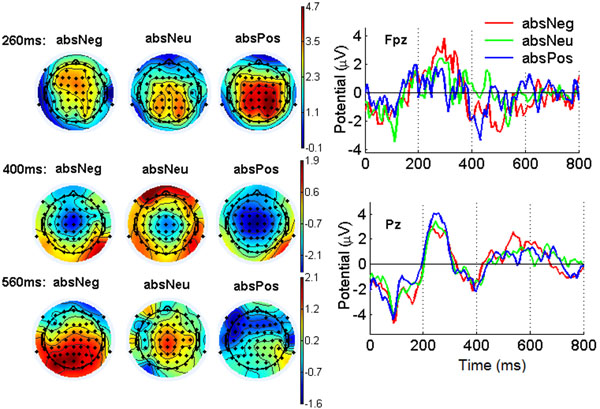
Grand mean ERPs elicited by abstract word stimuli. Left panel: ERP scalp maps at three time points of P300, N400 and P600 respectively; right panel: ERP waveform; at a prefrontal and a parietal EEG channel.

There has recently been a surge of interest in searching the neural basis of emotional connotations in words, using fMRI, MEG and EEG [1-4]. In this work we use a linguistically controlled set of 36 English word stimuli, where emotional valence and concreteness are cross-classified, and present them visually to five English native speakers (aged 26–39yrs, mean 34). EEG signals were recorded from the participants while they performed a mental simulation task. We use event-related potential (ERP) and multivariate pattern analyses (MVPA) to investigate the cortical representation and classification of emotional valence, for concrete words and abstract words separately. The grand mean ERPs elicited by abstract words over the five subjects show four typical ERP components: N100, P300, N400 and P600 (Fig. 1). For the P300 waveform, in prefrontal areas the amplitude of negative words is the largest, while that of positive words is the smallest; in contrast, in central-parietal-occipital regions the amplitude of positive words is the largest, whereas that of the negative words is the smallest. For the N400, the negative deflection of positive words is the largest, while that of the neutral words is the smallest in frontal-central areas. For P600, the amplitude of negative words is the largest in the posterior regions. For concrete words, the ERP patterns are broader, but with some important differences with regard to abstract words. The amplitude of P300 of negative words are closer to that of neutral words in the prefrontal area. There are no clear differences between negative, neutral and positive words during the P600. We then Use EEG amplitudes features extracted in the time-domain to train a sparse multinomial logistic regression (SMLR) classifier for three-way valence classification. The mean classification accuracy over the 5 subjects is 39.5% for abstract words and 41.0% for concrete words, which are above the chance level of 33.3%.
